# The Touch

**Published:** 1914-05

**Authors:** 


**A Monthly Review of Medicine and Surgery**

EDITOR AND PUBLISHER DR. A. L. BENEDICT, 228 Summer St., corner of Elmwood Ave., Buffalo, (Address for all communications. Please make personal and telephone calls before 1 P. M.)

Third, in age, on the western continent. Independent, but supports professional organization. News limited mainly to Western N. Y. and Penn.

Subscription $2.00 a year; $3.00 for two years in advance. Changes and errors in address or failure or duplication of delivery, should be reported immediately.

Yearly Volume 69 MAY, 1914 No. 10

### The Touch.

Within a comparatively few years, private hospitality, personal charity, friendly accommodation (which is usually in one direction and is later discovered to be a permanent donation), and the benevolences of the church, have been supplemented by a large number of systematic canvasses for money to carry on the most varied kinds of philanthropy, mostly worthy, but in some instances questionable, and in a few quite plainly involving the element of personal glory if not graft.

Some time ago, we kept track of invitations to contribute. They averaged ten dollars a week on the basis of a stingy response, twenty-five for the modest but adequate one for a poor man, with no assignable maximum. Then came a demand from the educational institution which, for system, multiplicity of objects and broadness of range of what it would like, easily wins first prize in mendicancy. This institution would cheerfully accept as a fairly liberal contribution to minor purposes, all we earn in a year; or the results of a couple of generations of self-denial and prudence, as a very minor donation to major purposes. At this point we ceased to gather statistics and put a rubber band around our check book.

At the risk of seeming to advocate heartlessness, we would remind the profession and especially the younger members, that the average professional income is meagre, that the physician receives no discounts from merchants and mechanics corresponding to what they ask of him, that in addition to intentional professional charity he does from fifty per cent. down to ten per cent. of his work free. In other words, the medical profession is rated popularly, in the various demands made upon it, at a much higher level financially than facts justify. It seems to us that, for the physician of average means, the normal church and personal benevolences which are the duty of every man, plus his strictly professional donations to philanthropy, plus a very few causes in which he is especially interested, should suffice.

Just a word about the tithe, which is being urged so strongly at present. As we understand it, the tithe of the Bible was a ten per cent. tax levied by the state and church, jointly, on the increase in capital for the year, after deducting ordinary living and business expenses, hospitality and minor personal charities. At present, the state, represented mainly by the municipality and county, appropriates 2% or more of the total valuation of capital, so far as it can be determined, amounting to approximately 15—30% of income from real property, probably much less from other forms of investment. In addition a considerable tax, difficult to estimate, is collected in various indirect ways. The present ecclesiastic attempt at tithing, is a levy of 10%, not on yearly increase after deducting reasonable expenses, but on the entire income, and for church purposes alone.

As we have stated, we do not mean to discourage liberality, but we think it would be well for our readers to consider certain good causes more or less competing with the demands made on them from a multiplicity of sources. For instance, there is a woman in black, very neat but a trifle shabby, calling on your colleagues, ten or twenty years from now, asking for bills to collect, or patients to nurse, or seeking roomers, or almost any kind of occupation calling for the knowledge which a good housekeeper has. The bills are already placed with a regular collector; younger and better trained women are caring for the patients; as to the rooming house, you know what it is like; yes there is a place for a matron or housekeeper, perhaps here, perhaps many miles away but, has she any children? Yes, two or three, but very well behaved. Unfortunately there are many practical reasons why she cannot take the children with her. There is the orphan asylum...... Oh, well, you know the case; it has been presented to you quite often; the problem is solved finally, in some way, sometimes pretty well, oftener very badly. You felt sorry in an impersonal way, but this woman looks like your own wife, grown older and more sorrowful, and you are not there to hear her story. That is one of the philanthropies which ought to be considered when you get a mimeographed letter asking for a day's or a month's earnings, with a receipt blank and addressed envelope inclosed. Another philanthropy is that of a girl, hurrying through a course in stenography, and then seeking a position about which you know a dozen salacious stories, not often but occasionally founded on fact. Still another is that of a boy, at a ten dollar a week job. He wanted to go to college but his father died, leaving a considerable circle of temporarily grieved patients, a rather imposing amount of bills due, which shrank in the collecting, a library worth rather more than a cent a pound, some instruments worth a little more, and a house too large for the family to maintain. And, if you are very selfish, there is another which strikes us as the saddest of all—a lonely old man, physically handicapped in a keen struggle for existence, perhaps still well equipped mentally, but so considered by pitifully few, hanging at the outskirts of his profession, living in penury, with nothing but memories to sustain him.

Weigh these philanthropies well against the various claims made upon you. Decide wisely, justly, and generously.

